# Effects of Hylan G-F 20 supplementation on cartilage preservation detected by magnetic resonance imaging in osteoarthritis of the knee: a two-year single-blind clinical trial

**DOI:** 10.1186/1471-2474-12-195

**Published:** 2011-08-24

**Authors:** Yuanyuan Wang, Stephen Hall, Fahad Hanna, Anita E Wluka, Gail Grant, Paul Marks, Marie Feletar, Flavia M Cicuttini

**Affiliations:** 1 Department of Epidemiology and Preventive Medicine, School of Public Health and Preventive Medicine, Monash University, Alfred Hospital, Melbourne, Australia; 2 Cabrini Health, Melbourne, Australia; 3 Department of Radiology, The Avenue Hospital Windsor, Melbourne, Australia

**Keywords:** Hyaluronic acid, Hylan, Cartilage, Magnetic resonance imaging (MRI), Osteoarthritis

## Abstract

**Background:**

Although viscosupplementation is an effective symptomatic treatment for knee osteoarthritis (OA), the effect of longer term administration on articular cartilage has not been fully explored. We examined the effect of viscosupplementation with Hylan G-F 20 on knee cartilage over 2 years in patients with knee OA.

**Methods:**

In this prospective, single-blind, parallel control group pilot study, 78 patients with symptomatic knee OA (Kellgren-Lawrence grade II and III) were assigned to either intervention group (n = 39 receiving 4 courses of 3 × 2.0 ml of intra-articular Hylan G-F 20 injections at 6 month intervals) or control group (n = 39 receiving usual care for knee OA without injections). Magnetic resonance imaging of the study knee was performed at baseline, 12 and 24 months. Cartilage volume and defects were assessed using validated methods.

**Results:**

Fifty-five subjects (71%) completed 24 month follow up. Over 24 months, the intervention group had a reduced annual percentage rate of medial and lateral tibial cartilage volume loss (mean ± SD, -0.3 ± 2.7% and -1.4 ± 4.3%) compared with the control group (2.3 ± 2.6% and 1.4 ± 2.6%, P = 0.001 and 0.005 for difference, respectively). The intervention group also showed reduced cartilage defect score increment in the medial tibiofemoral compartment (0.1 ± 1.3) compared with the control group (0.8 ± 1.5, P = 0.05).

**Conclusions:**

Six monthly intra-articular injections of Hylan G-F 20 administered to patients with symptomatic knee OA have a beneficial effect on knee cartilage preservation measured by both cartilage volume and cartilage defects. Hylan G-F 20 warrants further evaluation in larger clinical trials as a possible disease-modifying agent in the treatment of knee OA.

**Trial Registration:**

The study was registered with ClinicalTrials.gov (NCT00393393).

## Background

Osteoarthritis (OA), a major cause of pain and disability, results in significant morbidity and health care expense [1]. Whilst OA affects the whole joint, progressive cartilage degeneration characterises disease progression [2]. Despite its prevalence, current non-surgical treatments for OA relieve symptoms only; none are proven to have any disease-modifying effect. Hyaluronic acid is important in maintaining articular cartilage integrity, being one of the major glycosaminoglycans in the extracellular matrix. By binding proteoglycans, it provides and maintains intraarticular lubrication, optimising the viscoelastic properties of synovial fluid [3]. The concentration and molecular weight of hyaluronic acid in synovial fluid are reduced in osteoarthritic joints, increasing cartilage susceptibility to mechanical stress [4].

Intra-articular injections of different forms of hyaluronic acid are safe and effective in relieving pain and improving function in knee OA over the short to medium term [5,6]. Whilst intra-articular hyaluronic acid injection is registered as a device to treat symptomatic knee OA [5,6], evidence suggests that it may also retard disease progression and thus be a potential disease-modifying agent. In various animal models of knee OA, intra-articular hyaluronic acid inhibits the expression of interleukin-1 beta and metalloproteinase-3 in synovium, prevents proteoglycan content change in articular cartilage, inhibits articular cartilage degeneration, and reduces fibrosis and synovial vascularity [7-10]. In humans, small clinical trials employing arthroscopy with cartilage and synovial biopsies have suggested that hyaluronic acid administration may improve cartilage morphology, reduce synovial inflammation and cartilage deterioration over 6 months to 1 year [11-14]. However the data is conflicting regarding the effect of sodium hyaluronate on radiographic joint space narrowing over 1 year [14,15]. The only study examining the effect of intra-articular hyaluronic acid on articular cartilage assessed by magnetic resonance imaging (MRI) found no significant difference in patellofemoral cartilage changes between the treatment and control groups over 8 weeks [16]. However, the duration may be too short to detect differences in cartilage changes.

Hyaln G-F 20 is a cross-linked form of purified hyaluronan with high molecular weight, elastoviscous fluid with rheologic properties similar to the young healthy human synovial fluid in the knee. The study aimed to determine the effect of viscosupplementation on knee structural changes by examining the effect of repeated intra-articular injections of Hylan G-F 20 (Synvisc^®^) on the progression of cartilage changes assessed using MRI over 24 months in patients with symptomatic knee OA. The hypothesis was that hyaluronic acid viscosupplementation may preserve articular cartilage and retard the progression of knee OA.

## Methods

### Study design and participants

This was a prospective, single-blind, parallel control group pilot study performed in Melbourne, Australia. Patients were recruited from rheumatology and orthopaedic practices. Eligibility criteria were: age 18 to 80 years; symptomatic knee OA as defined by the American College of Rheumatology criteria [17]; Kellgren-Lawrence grade [18] of II or III on prior X-rays (taken within 6 months of the screen visit) or screen X-ray; knee pain score ≥40 mm on a 100 mm visual analogue scale for > 15 days in the last month. Oral/parenteral corticosteroids (≤10 mg/day prednisolone or equivalent) and NSAIDs were permitted if the dose had been stable for at least one month prior to baseline. Patients were excluded if they had unstable knee, a varus or valgus deformity of > 15 degrees; any contraindication to MRI; active malignancy; recent trauma (clinically defined) or known loose bodies in the study joint; inflammatory arthritis; concomitant medications of potent analgesics including opiates; oral or parenteral corticosteroid therapy within one month prior to enrolment into the study other than stable doses of ≤ 10 mg daily prednisolone or equivalent; previously received viscosupplementation therapy within 12 months of study enrolment; arthroscopic or open surgery within the previous 12 months or planned surgery to the study joint; intra-articular injection of corticosteroid to study joint within the past six months; morbid obesity defined as body mass index (BMI) ≥40 kg/m^2^; known sensitivity to any component of Synvisc; or an active systemic infection.

**Figure 1:**
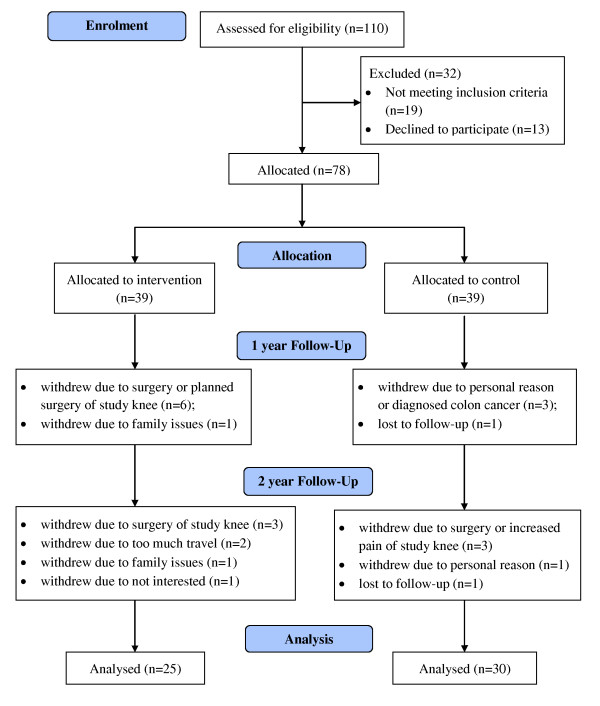
**Study profile**.

A total of 110 subjects were assessed for eligibility; 19 failed screening prior to initial MRI (ineligible), 13 withdrew from study prior to initiation of therapy for various reasons, generally withdrawal of consent. Therefore, 78 patients were assigned sequentially to either the intervention (n = 39) or the control group (n = 39) and had baseline MRI. Sixty-seven subjects attended the 12 month follow up as 11 withdrew from the study, and 55 subjects completed the 24 month follow up (Figure 1).

The study was approved by the Human Research Ethics Committee of Cabrini Health. All patients provided written informed consent before being assigned to study groups.

### Treatment

The intervention group received 4 courses of intra-articular Synvisc injections at 6 month intervals. Each course consisted of three intra-articular injections of 2.0 ml Synvisc administered into the study knee at weekly intervals. A parallel control group received no Synvisc treatment but received usual care for OA.

All other treatments received by the patients for knee OA remained stable for the duration of the study. This was monitored by asking the study subjects at each clinic visit if there had been any changes to their NSAIDs, analgesic and physical therapies throughout the study and documented in the study source notes. Acute Synvisc related flares of knee pain could be managed at the investigator's discretion with options including aspiration of the knee, analgesics and immobilisation. No subject received intra-articular corticosteroid injection.

### Anthropometric data

Height was measured using a stadiometer with shoes removed. Weight was measured with bulky clothing removed. BMI was calculated [weight (kg)/height^2 ^(m^2^)].

### Measurement of cartilage volume, defects, bone area, and bone marrow lesions (BMLs)

An MRI of the study knee of each participant was performed at baseline, 12 and 24 months. Knees were imaged in the sagittal plane on a 1.5-T whole body magnetic resonance unit (Signa Advantage HiSpeed; General Electric Medical Systems, Milwaukee, WI, USA) using a commercial transmit-receive extremity coil, with previously described sequences and parameters [19]. Each cartilage and bone measure was performed by one trained observer (F.H for cartilage volume, C.X for cartilage defects and tibial bone area, and M.D. for BMLs) with independent random cross checks performed by a second observer (Y.W), using the software Osiris (University Hospitals of Geneva, Switzerland) and ImageJ (National Institutes of Health, USA). All MRI images were analysed unpaired, blinded to patient group, data collected, and sequence.

Tibial cartilage volume and bone areas were measured using validated methods [19]. The coefficients of variation for the medial and lateral tibial cartilage volume measures were 3.4% and 2.0%, respectively [19]. The coefficients of variation for the medial and lateral tibial plateau bone area measures were 2.3% and 2.4%, respectively [19].

Cartilage defects were graded in the medial and lateral tibial and femoral cartilages [20]: grade 0, normal cartilage; grade 1, focal blistering and intracartilaginous low-signal intensity area with an intact surface and bottom; grade 2, irregularities on the surface or bottom and loss of thickness of < 50%; grade 3, deep ulceration with loss of thickness of ≥ 50%; grade 4, full-thickness cartilage wear with exposure of subchondral bone. If more than one cartilage defect was found in a cartilage plate, the highest grade was recorded. Intra- and inter-observer reliability (expressed as intraclass correlation coefficient) was 0.90 and 0.90 for the medial, 0.89 and 0.85 for the lateral tibiofemoral compartment, respectively [20]. The grades of tibial (0-4) and femoral (0-4) cartilage defects were added up as tibiofemoral cartilage defect score in the medial (0-8) and lateral (0-8) compartment, separately.

BMLs were defined as areas of increased signal intensity within the subchondral bone regions in distal femur or proximal tibia on coronal T_2_-weighted fat-saturated images [21]. The reproducibility for determination of BMLs was high (Kappa = 0.88, P < 0.001). A BML was defined as present if it appeared on two or more adjacent slices, encompassing at least one quarter of the width of medial or lateral compartment.

### Statistical analyses

With 39 subjects in each arm of the study, we had 80% power to show a 60% difference in the amount of knee cartilage loss over 2 years in the intervention group compared to the control group. This is based on our published MRI data of the amount of cartilage loss [22]. We anticipated a 2% annual cartilage volume loss in the intervention group compared to 5% in the control group (SD 5%). T-tests were used for comparison of means. Mann-Whitney U test was used to compare medians. Chi-squared test was used to compare nominal characteristics between the groups. The primary outcome measures were annual percentage change in tibial cartilage volume and change in cartilage defect score over time. The annual percentage change of cartilage volume was calculated by:

$$\frac{\text{initial}\;\text{volume}-\text{second}\;\text{volume}}{\left(\text{initial}\;\text{volume}\right)\left(\text{time}\;\text{between}\;\text{scans}\right)}\times 100\%$$

Change in tibiofemoral cartilage defect score was determined by subtracting baseline tibiofemoral defect score from follow-up tibiofemoral defect score. Outcome measures were initially assessed for normality before being regressed against exposure variables, thus general linear models were used to explore the relationship between intervention and outcome measures adjusting for potential confounders. All analyses were performed on completers. P < 0.05 (two-tailed) was regarded as statistically significant. All analyses were performed using the SPSS statistical package (version 16.0.0, SPSS, Cary, NC).

## Results

Seventy-eight patients were assigned to either the intervention (n = 39) or the control group (n = 39). There were no significant baseline differences between the two groups apart from the prevalence of medial tibiofemoral BMLs being higher in the control group compared with the intervention group (Table 1).

**Table 1:** Baseline characteristics of study population

	Intervention n = 39	Control n = 39	P value*
Age, years	61.3 (9.6)	61.4 (9.8)	0.97
Females, number (%)	19 (54)	14 (40)	0.23†
Body mass index, kg/m^2^	29.8 (3.7)	29.2 (4.8)	0.57
Kellgren-Lawrence grade, number (%)			0.43
2	28 (72)	31 (79)	
3	11 (28)	8 (21)	
Joint space narrowing, number (%)	38 (97)	38 (97)	1.00
Medial tibial cartilage volume, mm^3^	1512 (552)	1420 (624)	0.51
Lateral tibial cartilage volume, mm^3^	1691 (832)	1878 (685)	0.31
Medial tibial plateau bone area, mm^2^	2391 (400)	2537 (522)	0.20
Lateral tibial plateau bone area, mm^2^	1756 (335)	1841 (417)	0.35
Medial tibiofemoral cartilage defect score	5 [1, 8]	6 [1, 8]	0.15§
Lateral tibiofemoral cartilage defect score	3 [1, 8]	3 [0, 8]	0.32§
Prevalence of medial tibiofemoral bone marrow lesions, number (%)**	9 (38)	18 (72)	0.02†
Prevalence of lateral tibiofemoral bone marrow lesions, number (%)**	7 (29)	8 (32)	0.83†

Data were reported as mean (SD), median [range], or number (%)

*for difference between intervention and control groups using independent samples t test, Mann-Whitney U test§, or chi-squared test† where appropriate

**Bone marrow lesion data available in 24 subjects of intervention group and 25 subjects of control group who completed 24 month follow up

Sixty-seven (86%) subjects completed 12 month MRI follow up and 55 (71%) completed 24 month MRI follow up. The mean time between baseline and follow up MRI scans was 1.0 (SD 0.06, range 0.8-1.2) year and 2.2 (SD 0.2, range 1.9-3.2) years. There were no significant differences between those who completed the 12 month or 24 month follow up and those who did not in terms of age, gender, BMI, knee cartilage and bone area measures. The 55 completers were similar at baseline to the original 78 subjects. There were no significant differences in baseline characteristics of completers in the intervention (n = 25) and control (n = 30) groups, except that the control group had more prevalent medial tibiofemoral BMLs and tended to have greater lateral tibial cartilage volume, compared with the intervention group (Table 2). To examine the effect of intervention on cartilage changes over 12 and 24 months consistently and avoid the results being biased by any difference between the two subgroup populations (67 vs. 55 subjects), the following analyses were based on the 55 participants who completed both 12 and 24 month follow ups. However, similar results were observed when examining cartilage changes from baseline to 12 month follow up on the 67 participants (data not shown).

**Table 2:** Baseline characteristics of participants who completed 2 year follow up

	Intervention n = 25	Control n = 30	P value*
Age, years	61.6 (9.4)	61.3 (9.5)	0.89
Females, number (%)	15 (60)	12 (40)	0.14†
Body mass index, kg/m^2^	30.3 (3.9)	28.9 (4.5)	0.23
Kellgren-Lawrence grade, number (%)			0.10
2	16 (64)	25 (83)	
3	9 (36)	5 (17)	
Joint space narrowing, number (%)	24 (96)	29 (97)	0.90
Medial tibial cartilage volume, mm^3^	1488 (540)	1439 (619)	0.76
Lateral tibial cartilage volume, mm^3^	1485 (748)	1881 (720)	0.05
Medial tibial plateau bone area, mm^2^	2359 (415)	2503 (515)	0.27
Lateral tibial plateau bone area, mm^2^	1738 (325)	1827 (409)	0.38
Medial tibiofemoral cartilage defect score	4 [1, 8]	6 [1, 8]	0.08§
Lateral tibiofemoral cartilage defect score	4 [1, 8]	2.5 [0, 8]	0.23§
Prevalence of medial tibiofemoral bone marrow lesions, number (%)**	9 (38)	18 (72)	0.02†
Prevalence of lateral tibiofemoral bone marrow lesions, number (%)**	7 (29)	8 (32)	0.83†

Data were reported as mean (SD), median [range], or number (%)

*for difference between intervention and control groups using independent samples t test, Mann-Whitney U test§, or chi-squared test† where appropriate

**Bone marrow lesion data available in 24 subjects of intervention group and 25 subjects of control group who completed 24 month follow up

### Effect of intervention on change in tibial cartilage volume

From baseline to 12 months, the annual percentage changes in medial and lateral tibial cartilage volume were 1.4 ± 5.2% (P = 0.21, difference from zero change) and 2.8 ± 6.8% (P = 0.05), respectively for the intervention group, with significant loss in lateral tibial cartilage. In the control group, the annual percentage change was 3.3 ± 4.4% (P < 0.001) in the medial and1.0 ± 5.9% (P = 0.37) in the lateral tibial cartilage volume, with significant loss in medial tibial cartilage. However, there were no significant differences in annual percentage tibial cartilage volume changes between the intervention and control groups in either compartment (Table 3).

**Table 3:** The effect of intervention on annual percentage change in tibial cartilage volume

			Mean				Adjusted mean*	
	Intervention Mean (SD)	Control Mean (SD)	Difference† Mean (95% CI)	P value†	Intervention Mean (SE)	Control Mean (SE)	Difference† Mean (95% CI)	P value†
**From baseline to 12 month follow up**								
Medial tibial cartilage	1.4 (5.2)	3.3 (4.4)	1.9 (-0.7, 4.5)	0.15	1.3 (1.0)	3.3 (0.9)	2.0 (-0.8, 4.9)	0.16
Lateral tibial cartilage	2.8 (6.8)	1.0 (5.9)	-1.8 (-5.3, 1.6)	0.29	3.3 (1.3)	0.6 (1.1)	-2.7 (-6.2, 0.7)	0.12
**From baseline to 24 month follow up**								
Medial tibial cartilage	-0.3 (2.7)	2.3 (2.6)	2.6 (1.2, 4.1)	0.001	-0.4 (0.6)	2.4 (0.5)	2.7 (1.2, 4.3)	0.001
Lateral tibial cartilage	-1.4 (4.3)	1.4 (2.6)	2.8 (0.9, 4.7)	0.005	-1.3 (0.7)	1.3 (0.6)	2.6 (0.6, 4.5)	0.01

*Estimated marginal mean, adjusting for age, gender, body mass index, baseline tibial cartilage volume and bone area

†for difference between intervention and control groups

Over 24 months, whilst in the intervention group there was no significant annual percentage change in the medial (-0.3 ± 2.7%, P = 0.60) and lateral (-1.4 ± 4.3%, P = 0.12) tibial cartilage volumes, in the control group significant cartilage loss was seen in medial (2.3 ± 2.6%, P < 0.001) and lateral (1.4 ± 2.6%, P = 0.01) tibial cartilage volumes. The annual percentage cartilage loss was significantly higher in the control group compared with the intervention groups in both compartments (Table 3).

The results were similar after adjusting for age, gender, BMI, baseline tibial cartilage volume and bone area at both 12 and 24 months (Table 3). Adding the prevalence of BMLs in the models did not alter the findings (data not shown).

### Effect of intervention on change in tibiofemoral cartilage defects

From baseline to 12 months, the intervention group had a significant increase in cartilage defect score in the medial (0.4 ± 0.7, P = 0.02, difference from zero change) and lateral (0.2 ± 0.4, P = 0.04) compartments, while there was no significant change in cartilage defect score in the control group (0.1 ± 0.5, P = 0.26 and 0.2 ± 1.0, P = 0.28, respectively). However, the difference in change in cartilage defect score between the intervention and control groups was not statistically significant (Table 4).

**Table 4:** The effect of intervention on change in cartilage defect score from baseline to follow up

			Mean				Adjusted mean*	
	Intervention Mean (SD)	Control Mean (SD)	Difference† Mean (95% CI)	P value†	Intervention Mean (SE)	Control Mean (SE)	Difference† Mean (95% CI)	P value†
**From baseline to 12 month follow up**								
Medial tibiofemoral	0.4 (0.7)	0.1 (0.5)	-0.3 (-0.6, 0.1)	0.11	0.4 (0.1)	0.1 (0.1)	-0.3 (-0.6, 0.1)	0.15
Lateral tibiofemoral	0.2 (0.4)	0.2 (1.0)	0.04 (-0.4, 0.5)	0.85	0.2 (0.2)	0.2 (0.1)	-0.1 (-0.5, 0.4)	0.78
**From baseline to 24 month follow up**								
Medial tibiofemoral	0.1 (1.3)	0.8 (1.5)	0.8 (0, 1.5)	0.05	0.04 (0.3)	0.9 (0.3)	0.8 (0, 1.7)	0.05
Lateral tibiofemoral	0.4 (1.3)	0.8 (1.4)	0.4 (-0.4, 1.1)	0.33	0.5 (0.3)	0.7 (0.2)	0.2 (-0.5, 0.9)	0.56

*Estimated marginal mean, adjusting for age, gender, body mass index, and baseline cartilage defect score

†for difference between intervention and control groups

Over 24 months, while there was no significant change in cartilage defect score in the intervention group in the medial (0.1 ± 1.3, P = 0.75) or lateral (0.4 ± 1.3, P = 0.10) compartment, the control group had a significant increase in cartilage defect score in both compartments (0.8 ± 1.5, P = 0.005 and 0.8 ± 1.4, P = 0.003, respectively). The cartilage defect score in medial compartment in the control group increased significantly more than in the intervention group (P = 0.05) (Table 4).

The results were similar after adjusting for age, gender, BMI, and baseline cartilage defect score at both 12 and 24 months (Table 4). Adding the prevalence of BMLs in the models did not alter the findings (data not shown).

## Discussion

This prospective, single-blind, parallel control group pilot study reported the effect of repeated courses of Hylan G-F 20 on knee cartilage assessed using MRI over 2 years. The Hylan G-F 20 treated group showed reduced tibial cartilage volume loss and less increase in cartilage defect score compared with the control group over 24 months, independent of age, gender, BMI, and baseline cartilage and bone characteristics.

Although intra-articular hyaluronic acid injections have beneficial effects on articular cartilage and synovium assessed by arthroscopy [11-14], their effect on radiographic characteristics of knee OA has been inconsistent. While a small study of 36 patients showed no effect of the intervention on joint space narrowing reduction over 1 year [14], a randomised, placebo-controlled clinical trial in 273 patients found significantly less progression of joint space narrowing over 1 year associated with the intervention in patients with radiologically milder disease but not in those with radiologically more severe OA [15]. However, radiographic joint space narrowing is an indirect measurement of cartilage thinning and insensitive to change [23]. In contrast, MRI allows direct visualization of all components of the knee joint simultaneously and noninvasively, providing accurate quantification of articular cartilage which is sensitive to longitudinal change [24,25].

The only study examining the effect of intra-articular hyaluronic acid injections on articular cartilage assessed by MRI detected significant difference in patellofemoral cartilage defects over 8 weeks in the hyaluronic acid group, but it was not significant compared to the control group [16]. However, the small sample size (n = 30) and short time span of the study limited its ability to draw strong conclusion on the effect of intra-articular hyaluronic acid injections on articular cartilage [16]. We used a validated MRI protocol, providing accurate and reproducible quantitation of knee cartilage with 2 years follow up [24,25]. While at 12 months a trend towards less medial tibial cartilage loss was observed in the intervention group compared with the control group, at 24 months, intra-articular Hylan G-F 20 injections were associated with significantly reduced disease progression measured by both cartilage volume and defects: knee OA progression is associated with worsening of cartilage defects and reduced cartilage volume.

Cartilage volume loss measured from MRI is associated with worsening of knee symptoms [19] and increased risk of knee joint replacement [24]. Cartilage defects are associated with knee pain, cartilage breakdown, predict cartilage loss and knee joint replacement [20,25,26]. Thus we showed a beneficial effect of intra-articular Hylan G-F 20 injections on knee cartilage. Furthermore, the effect was more evident in the medial tibiofemoral compartment, the most common site of OA involvement at the knee. As the medial compartment is exposed to increased forces through the knee during weight-bearing activities [27] and increased joint loading plays a key role in the progression of knee OA [28], the medial compartment is more vulnerable to disease progression. Our findings suggest intra-articular Hylan G-F 20 may have the potential to retard the progression of knee OA and serve as a possible disease-modifying agent for OA which warrant further evaluation in larger clinical trials.

Although the mechanisms of the action of hyaluronic acid on articular cartilage still remain unclear, stimulation of endogenous hyaluronan and cartilage matrix synthesis, inhibition of cartilage degradation and inflammatory mediators may play a role [29,30]. The administration of repeat courses of sodium hyaluronate or Hylan G-F 20 over 1~2 years is safe and effective for pain relief in knee OA [31,32]. However, little information is available regarding the effect of repeat courses of sodium hyaluronate on knee structural changes [14,15]. In our study, the patients received 4 courses of Hylan G-F 20 every 6 months over 2 years where repeated intra-articular injections of Hylan G-F 20 reduced knee cartilage loss in both tibiofemoral compartments over 2 years with a trend suggesting reduced cartilage loss in the medial compartment over 1 year. It may be that we did not have power to show an effect over that shorter time period. The findings suggest that Hylan G-F 20 may have sustained effect on the preservation of knee cartilage and the effect is more evident with repeat administration over a longer time. Therefore repeated courses may be required to benefit cartilage. This will need to be examined in larger studies.

This study has limitations. Being a single-blind, parallel control group pilot study, participants were not randomly assigned to the intervention and control arms and no placebo control was performed. Although the baseline characteristics of the two groups were similar in terms of age, gender, BMI, knee cartilage and bone area measures, it is likely that other risk factors for OA progression such as knee malalignment and meniscal pathology, were different between the two groups. Although patients with unstable knee, a varus or valgus deformity of > 15 degrees were excluded from the study, we were not able to control for the confounding of those variables that we did not collect data on. The analysis was based on objective validated structural measures not subjective measures of symptoms. The presented analyses were based on the 55 of 78 (71%) of subjects who completed 24 month follow up and so will need to be interpreted with caution. Although the rate of loss to follow up was 29%, the 55 completers had similar baseline characteristics to the original 78 subjects, and the baseline characteristics of the 55 subjects in the intervention and control groups were similar. There were 9 patients in the treatment group who withdrew due to surgery or scheduled surgery of the study knee and therefore were not included in the final analysis (vs. 3 patients in the control group). This may have introduced selection bias that resulted in a better progression profile in the treatment group compared with the control group and therefore biased the results toward a protective effect of Hylan G-F 20. BMLs, a predictor of OA progression [21], were more prevalent in the control group compared with the treatment group. This may provide an alternative explanation of the increased OA progression we observed in the control group. However, BMLs were adjusted for in the analysis and the findings did not change. Randomized clinical trials will be needed to minimize selection bias and control for confounding. The strength of the study was the independently performed measurement of cartilage volume and defects, by independent experienced observers, who were blinded to both the intervention/control status of the participants and sequence of images.

## Conclusions

Although this is a pilot study with only moderate sample size, the findings suggest that 6 monthly intra-articular injections of Hylan G-F 20 administered to patients with symptomatic knee OA have beneficial effect on knee cartilage preservation, measured by both cartilage volume and cartilage defects. Over 24 months the control group continued to lose cartilage while there was no significant cartilage loss in the Hylan G-F 20 treated group. This use of Hylan G-F 20 warrants further evaluation in larger randomized, double-blind, placebo-controlled trials as a possible disease-modifying agent in the treatment of knee OA.

## Competing interests

The authors declare that they have no competing interests.

## Authors' contributions

YW was involved in data collection, performed data analysis and interpretation, and drafted the manuscript. SH contributed to the study design, oversaw the conduct of the trial, and was involved in data collection and interpretation. FH was involved in data collection and analysis. AEW was involved in data analysis and interpretation. GG and MF were involved in the conduct of the trial and data collection. PM oversaw imaging, provided technical support and was involved in data collection. FMC contributed to the study design, oversaw the measurement of knee structure, data analysis and interpretation, and coordinated all suggestions and edits. All authors participated in reviewing and editing the manuscript, and approved the final manuscript.

## Pre-publication history

The pre-publication history for this paper can be accessed here:

http://www.biomedcentral.com/1471-2474/12/195/prepub
